# WACPN: A Neural Network for Pneumonia Diagnosis

**DOI:** 10.32604/csse.2023.031330

**Published:** 2023

**Authors:** Shui-Hua Wang, Muhammad Attique Khan, Ziquan Zhu, Yu-Dong Zhang

**Affiliations:** 1 School of Computing and Mathematical Sciences, University of Leicester, Leicester, LE1 7RH, UK; 2 Department of Computer Science, HITEC University Taxila, Taxila, Pakistan

**Keywords:** wavelet entropy, community-acquired pneumonia, neural network, adaptive inertia weight factor, Rossler attractor, particle swarm optimization

## Abstract

Community-acquired pneumonia (CAP) is considered a sort of pneumonia developed outside hospitals and clinics. To diagnose community-acquired pneumonia (CAP) more efficiently, we proposed a novel neural network model. We introduce the 2-dimensional wavelet entropy (2d-WE) layer and an adaptive chaotic particle swarm optimization (ACP) algorithm to train the feed-forward neural network. The ACP uses adaptive inertia weight factor (AIWF) and Rossler attractor (RA) to improve the performance of standard particle swarm optimization. The final combined model is named WE-layer ACP-based network (WACPN), which attains a sensitivity of 91.87±1.37%, a specificity of 90.70±1.19%, a precision of 91.01±1.12%, an accuracy of 91.29±1.09%, F1 score of 91.43±1.09%, an MCC of 82.59±2.19%, and an FMI of 91.44±1.09%. The AUC of this WACPN model is 0.9577. We find that the maximum deposition level chosen as four can obtain the best result. Experiments demonstrate the effectiveness of both AIWF and RA. Finally, this proposed WACPN is efficient in diagnosing CAP and superior to six state-of-the-art models. Our model will be distributed to the cloud computing environment.

## 1 Introduction

Community-acquired pneumonia (CAP) is considered a sort of pneumonia [1] developed outside hospitals, and clinics, along with infirmaries [2]. CAP may affect people of any age, but it is more prevalent in very young and elderly groups, which may need hospital treatment if they develop CAP [3]. Chest computed tomography (CCT) is a crucial way to help radiologists/physicians to diagnose CAP patients. Recently, automatic diagnosis models based on artificial intelligence (AI) have gained promising performances and attracted researchers’ attention. For example, Heckerling, et al. [4] employed the genetic algorithm for neural networks to foresee CAP. This approach is shortened to the genetic algorithm for pneumonia (GAN). Afterward, Liu, et al. [5] proposed a computer-aided detection (CADe) model to uncover lung nodules in the CCT slides. Strehlitz, et al. [6] presented several prediction systems by means of support vector machines (SVMs) together with Monte Carlo cross-validation. Dong, et al. [7] proposed an improved quantum neural network (IQNN) for pneumonia image recognition. Ishimaru, et al. [8] proposed a decision tree (DT) model to foresee the atypical pathogens of CAP. Zhou [9] introduced the cat swarm optimization (CSO) method to recognize CAP. Wang, et al. [10] proposed an advanced deep residual dense network for the image super-resolution problem. Wang, et al. [11] proposed a CFW-Net for X-ray based COVID-19 detection.

However, the above methods still have room to improve. Their recognition performances, for example, the accuracies, are no more than or barely above 91.0%. We analyze their models and believe the reason is their training algorithms. After comparing recent global optimization algorithms, we find that particle swarm optimization (PSO) is one of the most successful optimization algorithms, compared to otheroptimization algorithms such as artificial bee colony [12] and bat algorithm [13]. Hence, we use the framework in Zhou [9] but replace CSO with an improved PSO. In addition, we introduce the two-dimensional wavelet-entropy (2d-WE) layer, introduce an improved PSO method—adaptive chaotic PSO (ACP) [14], and combine it with a feed-forward neural network. The final combined model is named WE-layer ACP-based network (WACPN). The experiments show the effectiveness of this proposed WACPN model. In all, we exhibit three contributions:

(a) The 2d-WE layer is managed as the feature extractor.
(b) ACP is utilized for training the neural network to gain a robust classifier.
(c) The proposed WACPN is proven to give better results than six state-of-the-art models.

## 2 Dataset and Preprocessing

**Figure 1:**
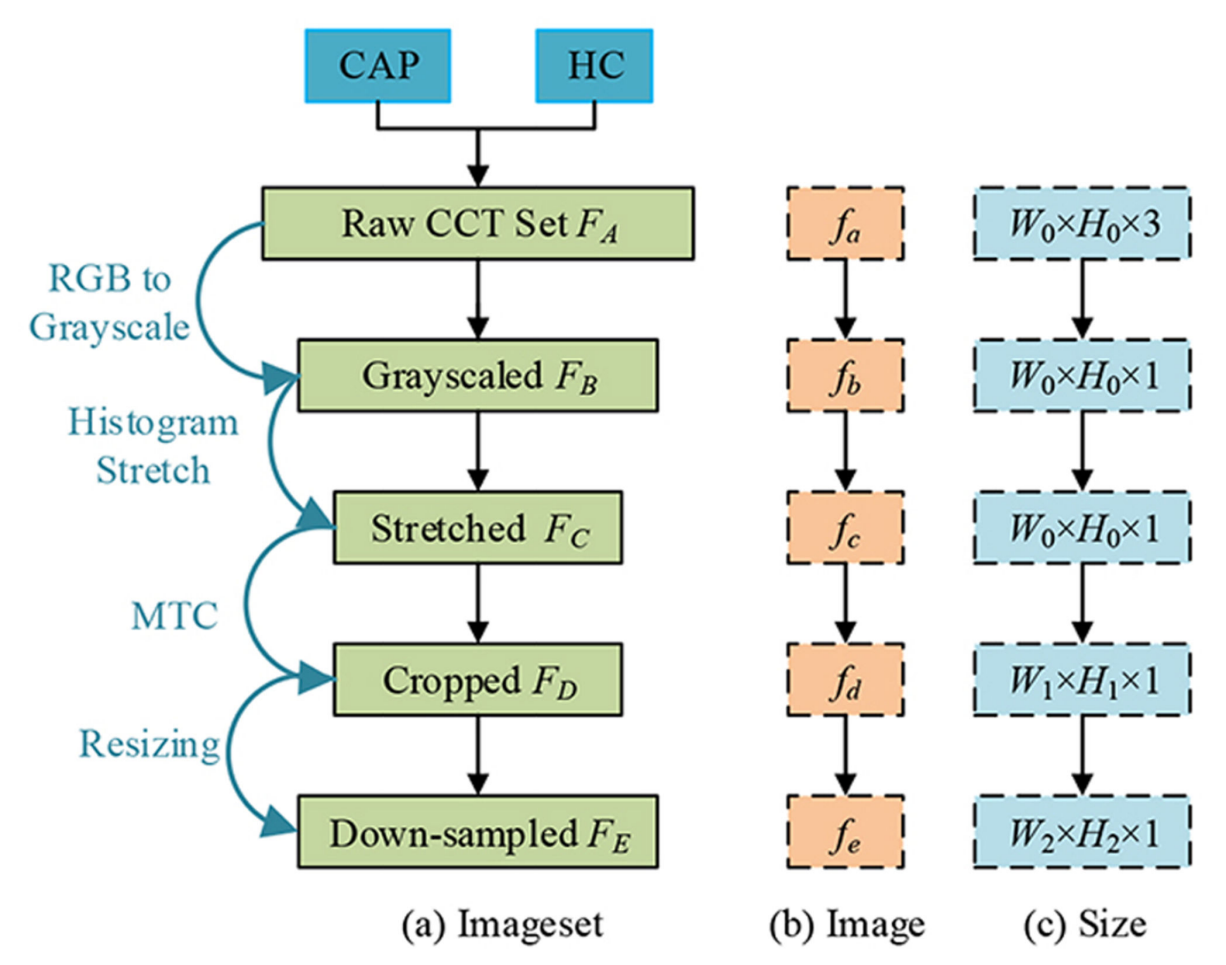
Diagram of preprocessing

The dataset is described in Zhou [9], where we have 305 CAP images and 298 healthy control (HC) images. The detailed demographical information can be found in Ref. [9]. Assume the raw CCT dataset is signified as *F_A_,* within which each image be signified as *f_a_,* and the number of entire images of both classes is |*F*| = 603, we get *F_A_* = {*f_a_*(*i*), *i* = 1,2, …, |*F*|}. The size of each image can be obtained as:

(1) $$h_{size}[f_{a}(i)]=W_{0}\times H_{0}\times 3,$$

where (*W*_0_,*H_0_)* connotes the width and height of the image set *F_A_* and *h_size_*(*x*) outputs the size of *x*. Here *W*_0_ = *H*_0_ = 1024. Fig. 1(a-b) depicts the schematic for preprocessing, which aims to grayscale the raw images, enhance their contrasts, cut the margins and texts, and resize the images.

Initially, the color CCT image set *F_A_* is transformed into grayscale images by holding the luminance channel. The grayscaled CCT image set is symbolized as *F_B_* = {*f_b_*(*i*), *i* = 1,2,…, |*F*|}.

Second, we use histogram stretching (HS) on all images *F_B_* = {*f_b_*(*i*)} to enhance the contrast. Take the *i*-th image *f_b_*(*i*) as a case, its image-wise minimum, and maximum grayscale value *f_b_^l^*(*i*) and *f_b_^h^*(*i*) are calculated as:

(2) $$\{\begin{array}{l} f_{b}^{l}(i)={\text{min}}_{p_{w}=1}^{W_{0}}{\text{min}}_{p_{h}=1}^{H_{0}}f_{b}(i|p_{w},p_{h})\\ f_{b}^{h}(i)={\text{max}}_{p_{w}=1}^{W_{0}}{\text{max}}_{p_{h}=1}^{H_{0}}f_{b}(i|p_{w},p_{h}) \end{array},$$

where (*p_w_, p_h_*) are temporary variables signifying the index of width and height along with the image *f_b_*(*i*), respectively. The HSed image set *F_c_* = {*f_c_*(*i*), *i* = 1, …, |*F*|} can be determined as:

(3) $$f_{c}(i)=\frac{f_{b}(i)-f_{b}^{l}(i)}{f_{b}^{h}(i)-f_{b}^{l}(i)}$$

Third, margin & text cropping (MTC) is implemented to eradicate (a) the checkup bed at the bottom zone, (b) the privacy-related scripts at the margin or corner zones, and (c) the ruler adjacent to the right-side and bottom zones. The MTCed image set *F_D_* = {*f_d_*(*i*), *i* = 1, …, |*F*|} can be determined as *f_d_*(*i*) = *f_c_*(*i; p_w_, p_h_*), *p_w_* ∈ [*p*_1_ + 1, *W*_0_ - *p*_2_], *p_h_* ∈ [*p*_3_ + 1, *H*_0_ - *p*_4_], where (*p*_1_, *p*_2_, *p*_3_, *p*_4_) stand for pixels to be cut from four directions (left, right, top, and bottom) with the unit of pixels. Note here the size of *f_d_*(*i*) is *h_size_*[*f_d_*(*i*)] = *W*_1_ × *H*_1_. By means of straightforward maths calculation, we reckon that

(4) $$\left\{ \begin{array}{l} W_{1}=W_{0}-p_{1}-p_{2}\\ H_{1}=H_{0}-p_{3}-p_{4} \end{array}\right.$$

Lastly, each image in *F_D_* is resized to the extent of [*W*_2_,*H*_2_], acquiring the resized image set *F_E_* = {*f_e_*(*i*), *i* = 1, …, |*F*|} as *f_e_*(*i*) = *h_resize_*[*f_d_*(*i*); (*W*_2_, *H*_2_)], where *h_resize_* signifies the resizing function.

**Figure 2:**
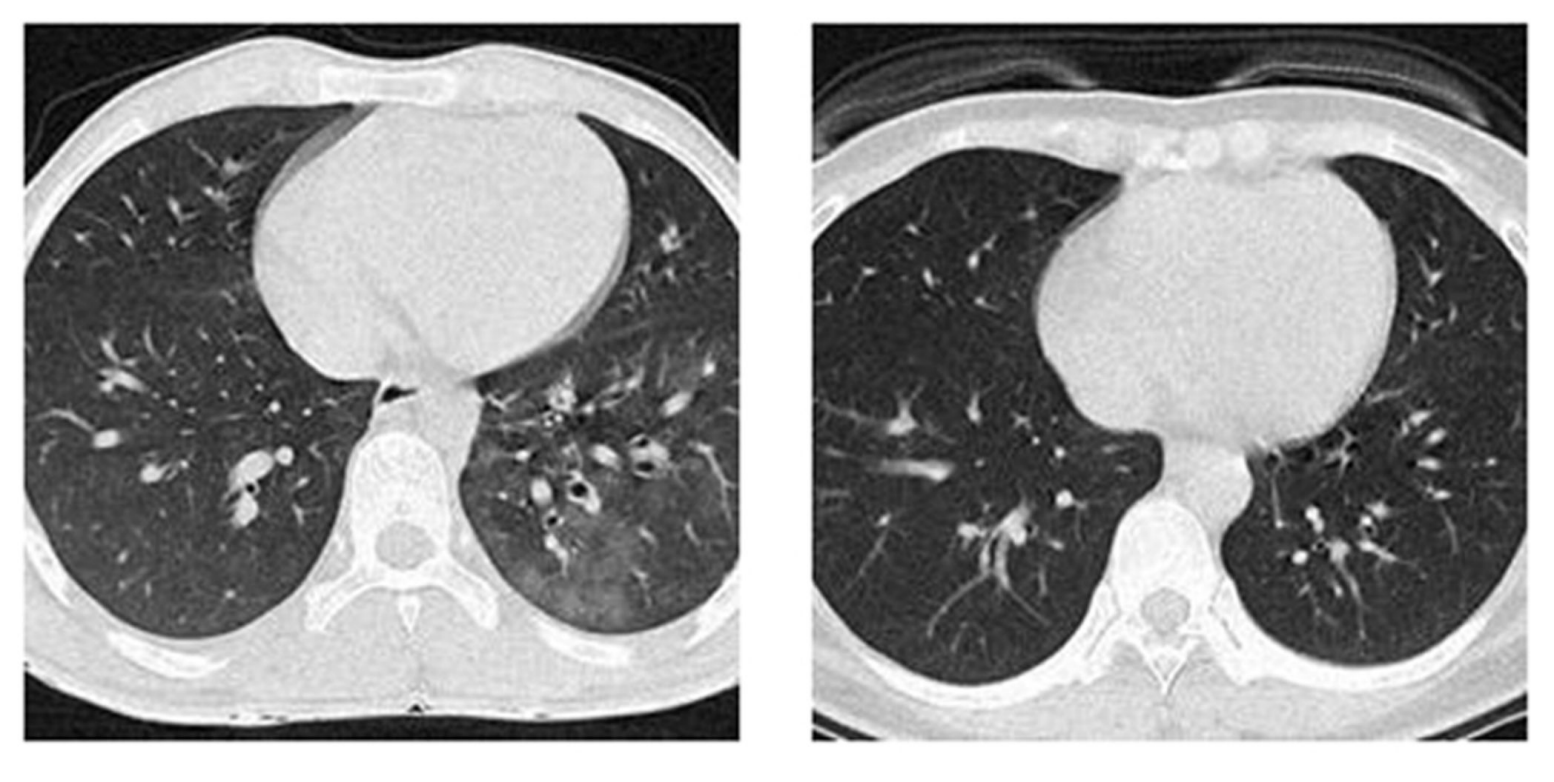
Examples of the preprocessed image set

Fig. 1(c) shows the extent of every raw image in *F_A_* is *W*_0_ × *H*_0_ × 3, and that of the final preprocessed image in *F_E_* is reduced to *W*_2_ × *H*_2_. In addition, the value of data-compression ratio (DCR) *z*_1_ is obtained as *z*_1_ = *W*_0_ × *H*_0_ × 3/(*W*_2_ × *H*_2_) = 48. The value of space-saving ratio (SSR) *z*_2_ is calculated as *z*_2_ = 1 — *W*_2_ × *H*_2_/(*W*_0_ × *H*_0_ × 3) = 97.92%. Fig. 2 shows two examples of the preprocessed image set. We use 10-fold cross-validation in our experiment.

## 3 Methodology of WACPN

### 3.1 Discrete Wavelet Transform

Tab. 1 enumerates all abbreviations and their associated meanings. The advantage of wavelet transform (WT) is that it holds both time/spatial and frequency information of the given signal/image. Nevertheless, the discrete wavelet transform (DWT) is chosen to convert the raw signal *r*(*t*) into the wavelet coefficient domain [15] in reality. Suppose the signal *r*(*t*) is one-dimension, first, we define the continuous wavelet transform (CWT) *E_γ_*(*s_a_, s_t_*) of *r*(*t*) as:

(5) $$E_{\gamma }(s_{a},s_{\tau })=\int_{-\infty }^{\infty }r(t)\times \gamma (t|s_{a},s_{t})dt,$$

in which *E* stands for the wavelet coefficient, *γ* the mother wavelet. *γ*(*t*|*s_a_, s_t_*) is defined as:

(6) $$\gamma (t|s_{a},s_{t})=\frac{1}{\sqrt{s_{a}}}\gamma (\frac{t-s_{t}}{s_{a}}),s_{a}>0\;,\;s_{t}>0,$$

where the *s_a_* signifies the scale factor (SF) and *s_t_* the translation factor (TF).

Now, we deduct the definition of DWT from CWT. The equation (5) is discretized by substituting *s_a_* and *s_t_* with two discrete variables (DVs) *c* and *v,*

(7) $$\left\{ \begin{array}{l} s_{a}=2^{c}\\ s_{t}=\nu \times 2^{c} \end{array}\right.$$

where *c* signifies the DV of the SF *s_a_*, and *v* the DV of the TF *s_t_* [16]. Moreover, the original signal *r*(*t*) is a DV to *r*(*q*), of which *q* signifies the DV of *t.* Like this, two subbands (SBs) can be calculated. The approximation SB *E^A^*(*q|c, v*) is determined as:

(8) $$E^{A}(q|c,\;v)=S_{D}[\sum_{o}r(q)\times f_{A}^{*}(\frac{q-2^{c}v}{2^{c}})],$$

where *f_A_*(*q*) signifies the low-pass filter. *S_D_* is the down-sampling operation. The detail SB *E^D^*(*q|c, v*) is determined as:

(9) $$E^{D}(q|c,\;\nu )=S_{D}[\sum_{o}r(q)\times f_{D}^{*}(\frac{q-2^{c}v}{2^{c}})]$$

where *f_D_*(*q*) signifies the high-pass filter.

### 3.2 2d-WE Layer

Suppose we handle a two-dimensional (2d) image *Q;* the 2d-DWT [17] is worked out by processing row-wise and column-wise 1d-DWT in succession [15]. Initially, the 2d-DWT operates on the original image *Q*. Later, four SBs (*Z*_1_, *O*_1_, *F*_1_, *A*_1_) are generated, where the subscript *i* means *i* -th level decomposition. Tab. 2 itemizes the description of four SBs. Note here MDL means the maximum decomposition level.

Assuming *h*^2*d-DWT*^ signifies a 2D-DWT decomposition operation, we deduce

(10) $$(\begin{array}{cc} A_{1} & Z_{1}\\ O_{1} & F_{1} \end{array})=h^{2d-DWT}(Q).$$

The subsequent decompositions run as:

(11) $$(\begin{array}{cc} A_{m} & Z_{m}\\ O_{m} & F_{m} \end{array})=h^{2d-DWT}(A_{m-1}),m=2,\;\ldots \;M,$$

where *M* is the MDL and *m* the current decomposition level [18].

**Figure 3:**
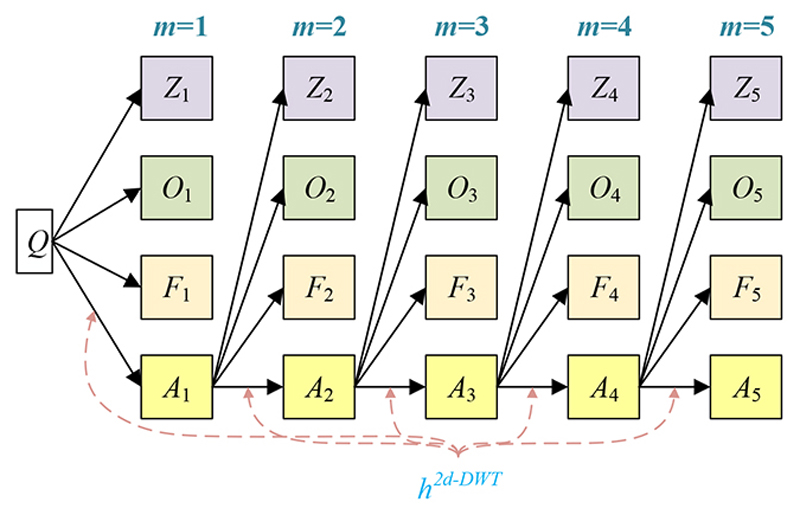
Diagram of a 2d-DWT (*M* = 5)

The subband *A*_1_ is further decomposed into four SBs (*A*_2_, *Z*_2_, *O*_2_, *F*_2_) at the 2nd level. The SB *A*_2_ is later decomposed to (*A*_3_, *Z*_3_, *O*_3_, *F*_3_), and then SB *A*_3_ is decomposed accordingly. Fig. 3 portrays a diagram of 5-level 2d-DWT, whose pseudocode is represented in Algorithm 1. This study chooses a *M-*level decomposition. The optimal value of *M* is found via trial-and-error approach [19] and related in Section 4.1.

Algorithm 1 **Pseudocode of 2d-DWT**

Input      Image *Q*

Step 1     Decompose the image *Q* into four subbands $\left(\begin{array}{cc} A_{1} & Z_{1}\\ O_{1} & F_{1} \end{array}\right)$.

                for *m* = 2: *M*

Step 2            The approximation subband *A*_*m*–1_ is decomposed into four subbands.

$A_{m-1}\overset{h^{2d-DWT}}{\mapsto }(\begin{array}{cc} A_{m} & Z_{m}\\ O_{m} & F_{m} \end{array})$

                end

Output   Output the (3*M* + 1) SBs (*A_M_, Z_M_, O_M_, F_M_*, *Z*_*M*–1_, *O*_*M*–1_, *F*_*M*–1_, …, *Z*_1_, *O*_1_, *F*_1_).

The (3*M* + 1) SBs (*A_M_, Z_M_, O_M_, F_M_*, *Z*_*M*–1_, *O*_*M*–1_, *F*_*M*–1_, …, *Z*_1_, *O*_1_, *F*_1_) may contain redundant features. Here we use the db4 wavelet. To decrease the number of features, we employ two-dimensional wavelet entropy (2d-WE) layer. The pseudocode of 2d-WE is illustrated in Algorithm 2. For each SB s in the generated (3*M* + 1) SBs, we imagine *s* to be a random DV S with *H* quantization values (*s*_1_, *s*_2_,..., *s_h_*,...,*s_H_*). In the beginning, we gauge the matching probability mass function (PMF) ***p***(*s*) = {*p_h_*(*s*)}.

(12) $$p_{h}(s)=h_{Pr}(S==s_{h}),h=1,2,\cdots \;H,$$

where *h_Pr_* signifies the probability function.

Second, the entropy of the PMF ***p***(*s*) is calculated as *f_e_*(*s*):

(13) $$f_{e}(s)=-\sum_{h=1}^{H}p_{h}(s)\times \text{ log }p_{h}(s),$$

where *f_e_* is the entropy function.

Lastly, the entropy values of the whole SBs are concatenated to grow a feature vector *I.*

(14) $$I=[\begin{array}{cccc} f_{e}(A_{M}) & f_{e}(Z_{M}) & f_{e}(O_{M}) & f_{e}(F_{M})\\ & f_{e}(Z_{M-1}) & f_{e}(O_{M-1}) & f_{e}(F_{M-1})\\ & \cdots & \cdots & \cdots \\ & f_{e}(Z_{1}) & f_{e}(O_{1}) & f_{e}(F_{1}) \end{array}],$$

where the number of the features in *I* is *N_I_*, = (3*M* + 1), which equals the number of SBs.

Algorithm 2 **Pseudocode of 2d-WE**

Input: (3*M* + SBs: *A_M_, Z_M_, O_M_, F_M_, Z_M–1_, O_M–1_, F_M–1_, …, Z_1_, O_1_, F_1_*)

for *m =* 1:3*M* + 1

       Choose the *m*-th SB *s*.

       Compute PMF ***p***(*s*) = {*p_h_*(*s*)}. See Eq. (12).

       Reckon entropy *f_e_*(*s*). See Eq. (13).

       Record *I*(*n*) ← *f_e_*(*s*).

End

Output: The concatenated 2d-WE feature vector *I* with *N_I_* features. See Eq. (14).

### 3.3 ACP Network

**Figure 4:**
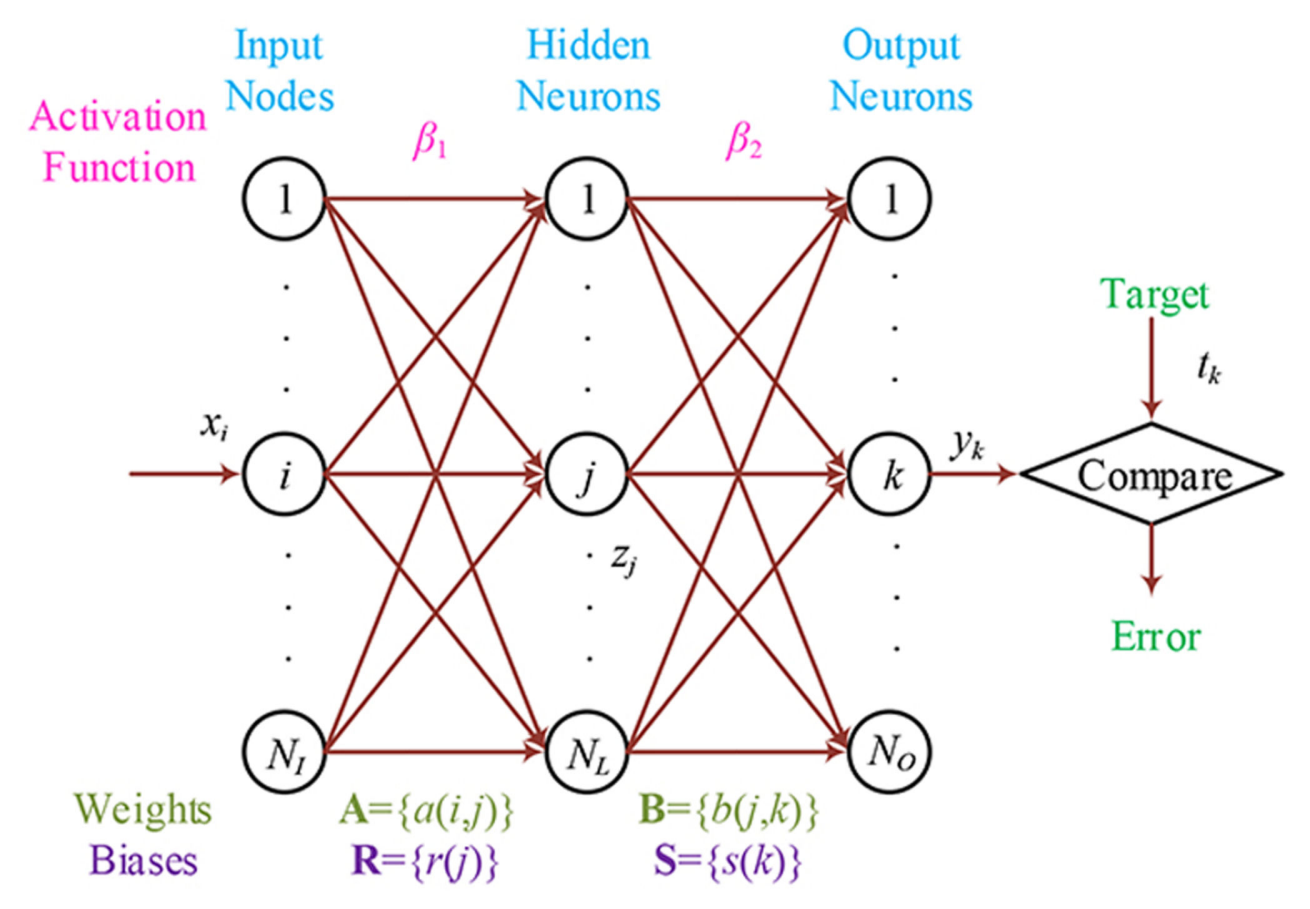
Diagram of an FNN

The *N_I_* features are thrown into a feed-forward neural network (FNN)—in which its inner connections do not make a loop. One-hidden-layer FNN (OFNN), represented in Fig. 4, is established due to the universal approximation theory. Assume (*x, t*) stands for a training case as: *x* = [*x*_1_, *x*_2_,..., *x_i_*,..., *x_N_i__*]^T^ signifies the input feature vector with *N_I_*-dimension, *i* denotes the neuron index at the input layer, *t* is the corresponding target label *t* = [*t*_1_, *t*_2_, …, *t_k_*, …, *t_N_O__*,]^T^, where *N_o_* signifies the number of prediction categories and *k* the node index at the output layer.Assuming *n* is the case index and *N* the number of entire training cases, this study symbolizes the training case (*x, t*) as {*x*(*n*), *t*(*n*)|*n* = 1,, …, *N*}. The training of the weights/biases (WBs) of OFNN is considered an optimization problem that minimizes the loss between the target *t* and the real output *y*. This study chooses the loss as the sum of the mean-squared error (MSE) *E* :

(15) $$E=\sum_{n=1}^{N}\sum_{k=1}^{N_{O}}{\left[y_{k}(n)-t_{k}(n)\right]}^{2}.$$

Assume *β*_2_ is the activation function (AF) in the output layer, and (**B, S**) are the WBs of neurons that connect the hidden layer (HL) to the output layer. **B** = {*b*(*j,k*)}, *j* = 1, ..., *N_L_*, *k* = 1, ... *N_O_*, and **S** = {*s*(*k*)}, *k* = 1, ..., *N_O_*. It is easy to reckon the output *y_k_* as

(16) $$y_{k}(n)=\beta_{2}[\sum_{j=1}^{N_{L}}b(j,k)z_{j}(n)+s(k)],$$

where *Z_j_*(_*n*_),_*j*_ = 1,..., *N_H_* signifies the output of *j*-th neuron in the HL. The description of *z_j_*(*n*) is

(17) $$z_{j}(n)=\beta_{1}[\sum_{i=1}^{N_{I}}a(i,j)x_{i}(n)+r(j)].$$

where **A** = {*a*(*i,j*)},*i* = 1, ...,*N_I_,j* = 1, ...,*N_L_* and **R** = {*r(j)*},*j* = 1, ...,*N_L_* are the WBs of the neurons that connect the input layer with the HL, and *β*_1_ the AF linked to the HL.

The parameter training is an optimization problem that guides us to search for the optimal WB parametric vector *θ =* (**A, B, R, S**). The length of *θ* is the number of parameters we need to optimize and is calculated as *N_θ_*=*N_I_*×*N_L_*+*N_L_*×*N_o_*+*N_L_*+*N_o_*. The training algorithm we choose is adaptive chaotic PSO (ACP) [14].

Recap that two attributes (position *x* and velocity *v)* are linked with each particle *p* in the standard PSO algorithm. Those two attributes are defined as the position of the particle (PoP) and the velocity of the particle (VoP). In each epoch, the fitness function *E* is re-calculated for the entire particles {*p*} in the swarm. The VoP *v* is re-evaluated by keeping track of the two best positions (BPs).

The first is the BP a particle *p* has traversed till now. It is dubbed *pBest* and symbolized as *x_pB_*. The second is the BP that any neighbor of *p* has traversed till now. It is a neighborhood best and is named *nBest* and symbolized as *x_nB_*.

If *p* takes the entire swarm as its neighborhood, the *nBest* turns to the global best and is for that reason named *gBest*. In standard PSO, the VoP *v* of particle *p* is updated as:

(18) $$v\leftarrow \omega v+b_{1}r_{1}(x_{PB}-x)+b_{2}r_{2}(x_{nB}-x)$$

where *ω* signifies the inertia weight (IW) controlling the influence of the preceding velocity of the particle on its present one. *b*_1_ and *b*_2_ stand for two positive constants named acceleration coefficients. *r*_1_ and *r*_2_ mean two random numbers, uniformly distributed in the range of [0,1]. *r*_1_ and *r*_2_ are re-calculated whenever they occur. The PoP *x* of the particle *p* is updated as:

(19) x←x+νΔt

where Δ*t* is the assumed time step and always equals 1 for simplicity.

The ACP algorithm proposed an adaptive IW factor (AIWF) strategy. It uses *ω_AIWF_* to replace *ω*.

(20) $$\omega_{AIWF}=\omega_{\text{ max }}-\frac{\omega_{\text{ max }}-\omega_{\text{ min }}}{k_{\text{ max }}}\times k$$

Here, *ω_max_* signifies the maximum IW, *ω_min_* the minimum IW, *k_max_* the epoch once the IW goes to the final minimum IW, and *k* the present epoch.

**Figure 5:**
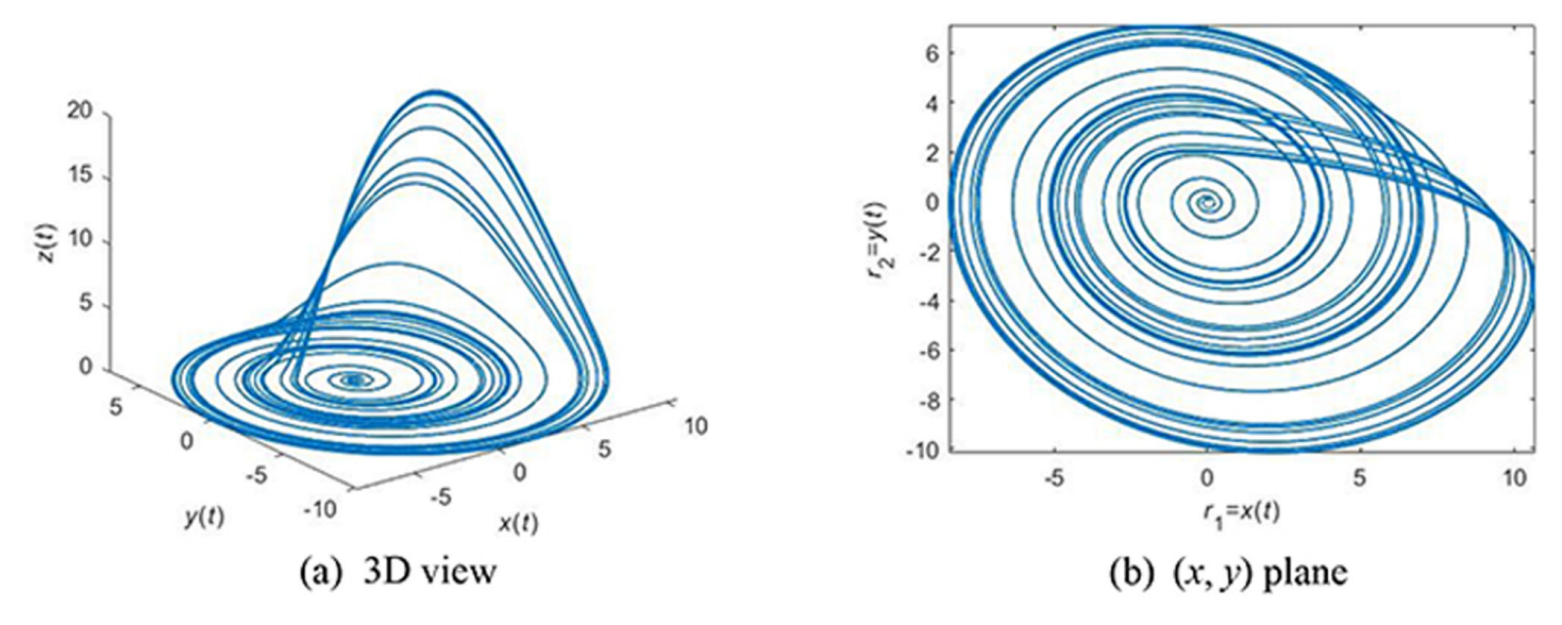
An example of RA with parameters of (*δ_a_* = 0.2, *δ_b_* = 0.4, *δ_c_* = 5.7)

Another improvement in ACP is upon the two random numbers (*r*_1_, *r*_2_). In reality, (*r*_1_, *r*_2_) are created by pseudo-random number generators (RNG), which cannot guarantee the optimization’s ergodicity in solution space since they are pseudo-random. Rossler attractor (RA) is a good choice to calculate the random numbers (*r*_1_, *r*_2_). RA equations are defined:

(21) $$\{\begin{array}{l} \frac{\text{d}x}{\text{d}t}=-(y+z)\\ \frac{\text{d}y}{\text{d}t}=x+\delta_{a}y\;\;,\\ \frac{\text{d}z}{\text{d}t}=\delta_{b}+xz-\delta_{c}z \end{array}$$

where *δ_a_, δ_b_,* and *δ_c_* are inherent parameters of RA. We choose *δ_a_* = 0.2, *δ_b_* = 0.4, *δ_c_* = 5.7 via the trial-and-error method [20]. The corresponding curve is drawn in Fig. 5(a). We agree *r*_1_ = *x*(*t*) and *r*_2_ = *y*(*t*) to implant the chaotic properties of RA into the two parameters (*r*_1_,*r*_2_) in standard PSO. The (*x,y*) plane of RA is displayed in Fig. 5(b).

## 4 Experiments, Results, and Discussions

Ten runs of 10-fold cross-validation are used to relate a reliable performance of our WACPN model. Besides, we use the following measures—sensitivity (Sen, symbolized as *η*_1_), specificity (Spc, symbolized as *η*_2_), precision (Prc, symbolized as *η*_3_), accuracy (Acc, symbolized as *η*_4_), F1 score (symbolized as *η*_5_), Matthews correlation coefficient (MCC, symbolized as *η*_6_), Fowlkes-Mallows index (FMI, symbolized as *η*_7_), and the area under the curve (AUC)—to appraise the performances of different models.

### 4.1 Parameter Configuration

The parameters of this study are listed in Tab. 3. The sizes of the original images are 1024 × 1024 if we do not consider the number of color channels. The sizes of MTCed images are 624 × 624, and the sizes of preprocessed images are 256 × 256. The DCR is *z*_1_ = 48, and the SSR is *z*_2_ = 97.92%. The MDL is *M* = 4. The number of features is *N_I_* = 13. The number of neurons in HL is *N_L_* = 8. The number of output neurons is *N_o_* = 2. The number of parameters to be optimized is *N_θ_* = 130. The parameters in RA are *δ_a_* = 0.2, *δ_b_* = 0.4, *δ_c_* = 5.7.

### 4.2 Wavelet Decomposition

**Figure 6:**
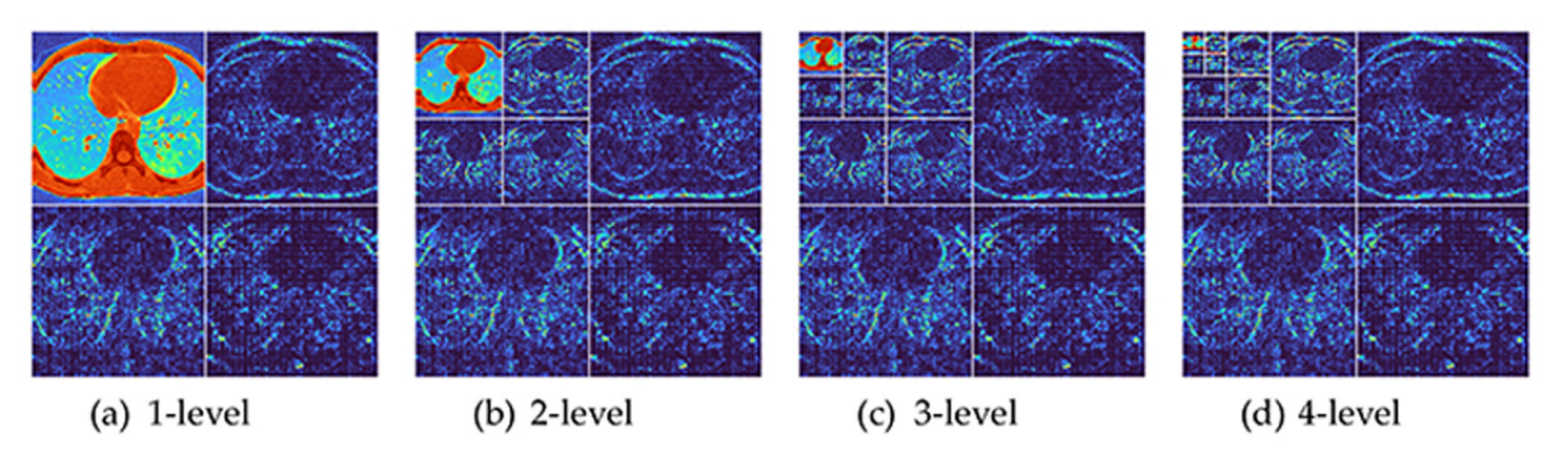
Wavelet decomposition results

Fig. 6 shows the wavelet decomposition results with *M* = 4. The raw image is shown in Fig. 2(a). The reason why we choose *M* = 4 is the trial-and-error method. We test other values of *M* and find *M* = 4 can obtain the best result.

### 4.3 Results of Proposed WACPN Model

Tab. 4 shows the ten runs of 10-fold CV via the parameters shown in Tab. 3, where *p_r_* = 1,2.,..,10 means the run index. The final row in Tab. 4 presents the mean and standard deviation (MSD) of the results of 10 runs. WACPN attains a sensitivity of 91.87±1.37%, a specificity of 90.70±1.19%, a precision of 91.01±1.12%, an accuracy of 91.29±1.09%, an F1 score of 91.43±1.09%, an MCC of 82.59±2.19%, and an FMI of 91.44±1.09%.

### 4.4 Effects of AIWF and RA

If we remove the AIWF from our WACPN model, the results using the same configuration are shown in Tab. 5. Similarly, the results of removing RA from our WACPN model are shown in Tab. 6. After comparing the results in Tab. 4 against the results in Tab. 5 and Tab. 6, we can deduce that both strategies—AIWF and RA—are beneficial to our WACPN model.

**Figure 7:**
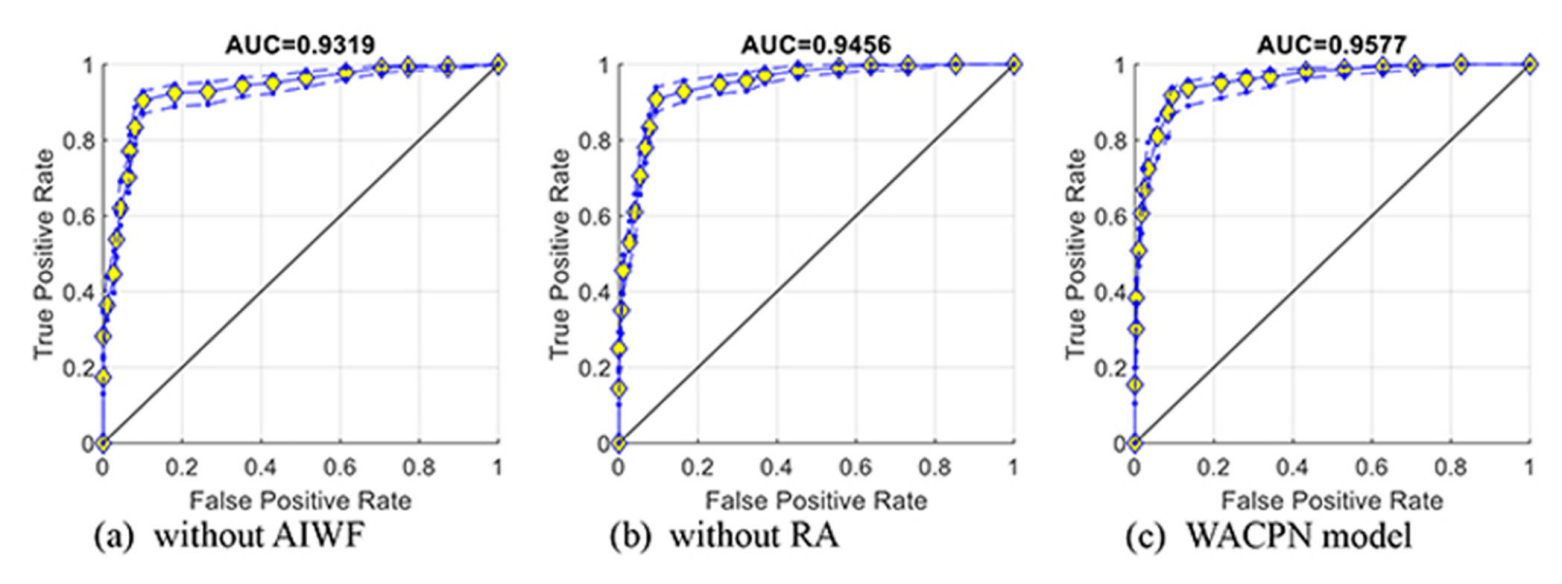
ROC curves

Fig. 7 represents the ROC curves together with their upper and lower bounds of the proposed WACPN model and its two ablation studies (without AIWF and without RA). The AUC of WACPN model is 0.9577. The AUCs of the models removing AIWF or RA are only 0.9319 and 0.9456, respectively, demonstrating that both AIWF and RA help improve the standard PSO.

### 4.5 Comparison with State-of-the-Art Models

The proposed WACPN model is judged with six state-of-the-art models: GAN [4], CADe [5], SVM [6], IQNN [7], DT [8], and CSO [9]. The evaluation results on the same dataset via ten runs of 10-fold CV are listed in Tab. 7.

**Figure 8:**
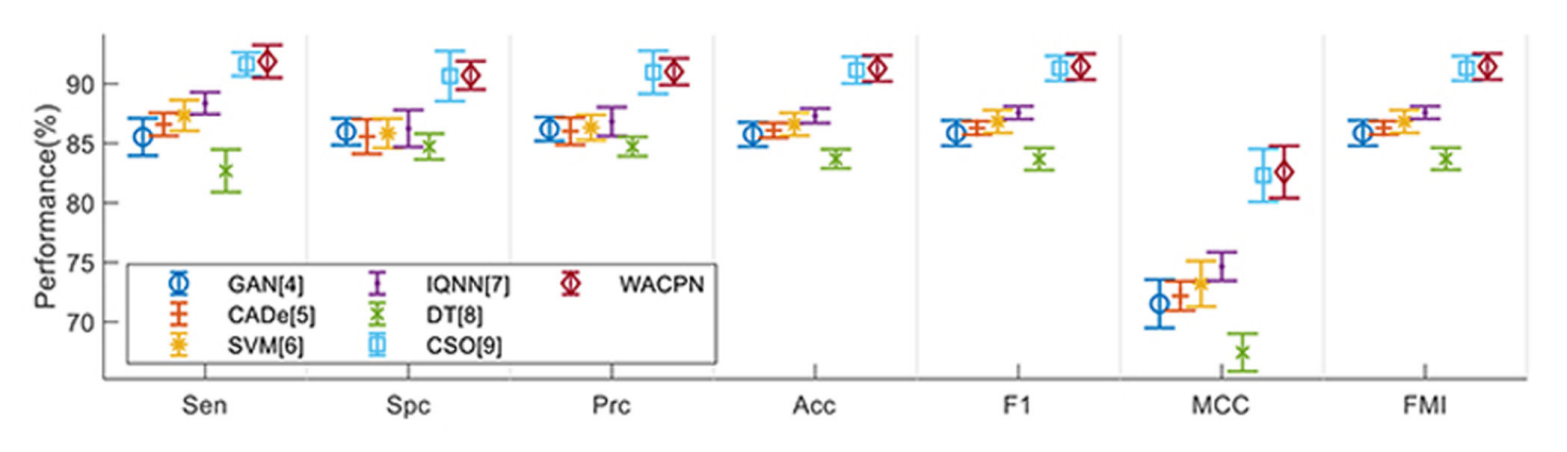
EB of model comparison

Error bar (EB) is an excellent tool for ease of visual evaluation. Fig. 8 presents the EB of model comparison, from which we can observe that the proposed WACPN model is superior to six state-of-the-art models. The causes are triple. First, the 2d-WE layer stands as a proficient way to designate CCT images. Second, ACP is efficient in training FNN. Third, we fine-tune and select the best parameters for the RA. In the future, our model may be applied to other fields [21, 22].

## 5 Conclusions

A novel WACPN method is proposed for diagnosing the CAP in CCT images. In WACPN, the 2d-WE layer works as feature extraction, and the optimization algorithm—ACP—is exercised to optimize the neural network. This proposed WACPN model is verified to have better results than six state-of-the-art models.

Three defects of the proposed WACPN model exist: (i) Deep learning models are not exercised. The reason is the small amount of our image set. (ii) Strict clinical validation is not tested either on-site or in cloud computing (CC) environments. (iii) The model is a black box, which does not go well with patients and doctors.

To work out the three limitations, first, we shall utilize the data augmentation method to enlarge the number of images in the dataset. Second, our team shall circulate the proposed WACPN model to the online CC environment (such as Azure) and summon specialists, clinicians, and physicians to examine its efficiency. Third, trustworthy or explainable Ais, which may provide the heatmaps pointing out the lesions, are two optional models to assist in adding explainability to the proposed WACPN model.

## Figures and Tables

**Table 1:** Abbreviation and meaning

Abbreviation	Meaning	Abbreviation	Meaning
2d-DWT	two-dimensional DWT	HS	histogram stretch
2d-WE	two-dimensional wavelet entropy	IW	inertia weight
ACP	adaptive chaotic PSO	MCC	Matthews correlation coefficient
AF	activation function	MDL	maximum decomposition level
AI	artificial intelligence	MSD	mean and standard deviation
AIWF	adaptive IW factor	MSE	mean-squared error
AUC	area under the curve	MTC	margin & text cropping
BP	best position	OFNN	one-hidden-layer FNN
CAP	community-acquired pneumonia	PoP	position of particle
CC	cloud computing	PMF	probability mass function
CV	cross-validation	PSO	particle swarm optimization
CWT	continuous WT	RA	Rossler attractor
DCR	data-compression ratio	SB	subband
DWT	discrete WT	SF	scale factor
DV	discrete variable	SSR	space-saving ratio
EB	error bar	TF	translation factor
FMI	Fowlkes-Mallows index	WACPN	WE-layer ACP-based network
FNN	feed-forward neural network	WB	weight and bias
GAN	genetic algorithm for pneumonia	WT	wavelet transform
HC	healthy control	VoP	velocity of particle
HL	hidden layer		

**Table 2:** Definition of four SBs

Symbol	Meaning	Symbol	Meaning
*Q*	Original image	*F*	Diagonal quadrant
*Z*	Horizontal quadrant	*A*	Approximate component quadrant
*O*	Vertical quadrant	*M*	MDL

**Table 3:** Parameter Setting

Parameter	Value	Parameter	Value
(*W*_0_,*H*_0_)	(1024,1024)	*M*	4
(*p*_1_, *p*_2_, *p*_3_, *p*_4_)	200	*N_I_*	13
(*W*_1_,*H*_1_)	(624,624)	*N_L_*	8
(*W*_2_,*H*_2_)	(256,256)	*N_O_*	2
*z*_1_	48	*N_θ_*	130
*z*_2_	97.92%	(*δ_a_*, *δ_b_*, *δ_c_*)	(0.2, 0.4, 5.7)

**Table 4:** Ten-run results of the proposed WACPN model

*p_r_*	*η*_1_	*η*_2_	*η*_3_	*η*_4_	*η*_5_	*η*_6_	*η*_7_
1	93.44	91.28	91.64	92.37	92.53	84.75	92.54
2	92.13	93.29	93.36	92.70	92.74	85.41	92.74
3	91.15	89.93	90.26	90.55	90.70	81.09	90.70
4	93.77	90.27	90.79	92.04	92.26	84.12	92.27
5	90.82	90.94	91.12	90.88	90.97	81.76	90.97
6	89.84	89.93	90.13	89.88	89.98	79.77	89.98
7	91.48	90.27	90.58	90.88	91.03	81.76	91.03
8	91.48	89.26	89.71	90.38	90.58	80.77	90.59
9	93.77	91.95	92.26	92.87	93.01	85.75	93.01
10	90.82	89.93	90.23	90.38	90.52	80.76	90.52
MSD	91.87±1.37	90.70±1.19	91.01±1.12	91.29±1.09	91.43±1.09	82.59±2.19	91.44±1.09

**Table 5:** Ten-run results without AIWF

*p_r_*	*η*_1_	*η*_2_	*η*_3_	*η*_4_	*η*_5_	*η*_6_	*η*_7_
1	89.51	90.60	90.70	90.05	90.10	80.11	90.10
2	89.84	86.58	87.26	88.23	88.53	76.47	88.54
3	89.84	90.27	90.43	90.05	90.13	80.10	90.13
4	90.49	87.92	88.46	89.22	89.47	78.45	89.47
5	89.18	90.27	90.37	89.72	89.77	79.44	89.77
6	91.48	87.58	88.29	89.55	89.86	79.15	89.87
7	93.44	93.29	93.44	93.37	93.44	86.73	93.44
8	89.51	89.93	90.10	89.72	89.80	79.44	89.80
9	92.46	92.62	92.76	92.54	92.61	85.07	92.61
10	88.52	88.59	88.82	88.56	88.67	77.11	88.67
MSD	90.43±1.56	89.77±2.15	90.06±1.96	90.10±1.63	90.24±1.58	80.21±3.25	90.24±1.58

**Table 6:** Ten-run results without RA

*p_r_*	*η*_1_	*η*_2_	*η*_3_	*η*_4_	*η*_5_	*η*_6_	*η*_7_
1	94.43	94.97	95.05	94.69	94.74	89.39	94.74
2	89.84	87.58	88.10	88.72	88.96	77.45	88.97
3	93.44	90.94	91.35	92.21	92.38	84.43	92.39
4	90.16	91.61	91.67	90.88	90.91	81.77	90.91
5	89.51	91.95	91.92	90.71	90.70	81.46	90.71
6	87.54	87.25	87.54	87.40	87.54	74.79	87.54
7	91.15	87.92	88.54	89.55	89.82	79.13	89.83
8	89.84	89.60	89.84	89.72	89.84	79.43	89.84
9	90.16	88.59	89.00	89.39	89.58	78.77	89.58
10	93.44	94.30	94.37	93.86	93.90	87.73	93.91
MSD	90.95±2.16	90.47±2.75	90.74±2.59	90.71±2.29	90.84±2.24	81.44±4.58	90.84±2.24

**Table 7:** Results of proposed WACPN and SOTA models (Unit: %)

Model	*η*_1_	*η*_2_	*η*_3_	*η*_4_	*η*_5_	*η*_6_	*η*_7_
GAN[4]	85.54±1.57	85.97±1.12	86.20±1.00	85.75±1.02	85.86±1.07	71.52±2.03	85.86±1.07
CADe[5]	86.59±0.96	85.57±1.45	86.02±1.14	86.09±0.62	86.29±0.56	72.18±1.23	86.30±0.56
SVM[6]	87.34±1.29	85.84±1.23	86.33±1.06	86.60±0.95	86.83±0.95	73.21±1.91	86.83±0.95
IQNN[7]	88.36±0.92	86.24±1.55	86.82±1.21	87.31±0.61	87.57±0.54	74.65±1.20	87.58±0.53
DT[8]	82.69±1.79	84.73±1.08	84.73±0.81	83.70±0.80	83.68±0.93	67.44±1.58	83.70±0.92
CSO[9]	91.64±0.99	90.64±2.11	90.96±1.81	91.14±1.12	91.29±1.04	82.31±2.22	91.29±1.03
WACPN	**91.87±1.37**	**90.70±1.19**	**91.01±1.12**	**91.29±1.09**	**91.43±1.09**	**82.59±2.19**	**91.44±1.09**

(Bold means the best)
